# The Hepatoprotective and MicroRNAs Downregulatory Effects of Crocin Following Hepatic Ischemia-Reperfusion Injury in Rats

**DOI:** 10.1155/2017/1702967

**Published:** 2017-03-06

**Authors:** Ghaidafeh Akbari, Seyyed Ali Mard, Mahin Dianat, Esrafil Mansouri

**Affiliations:** ^1^Physiology Research Center (PRC), Department of Physiology, School of Medicine, Ahvaz Jundishapur University of Medical Sciences, Ahvaz, Iran; ^2^Physiology Research Center (PRC), Research Center for Infectious Diseases of Digestive System, Department of Physiology, School of Medicine, Ahvaz Jundishapur University of Medical Sciences, Ahvaz, Iran; ^3^Cellular and Molecular Research Center, Department of Anatomical Sciences, School of Medicine, Ahvaz Jundishapur University of Medical Sciences, Ahvaz, Iran

## Abstract

*Background*. Liver ischemia-reperfusion (IR) injury is one of the chief etiologies of tissue damage during liver transplantation, hypovolemic shock, and so forth. This study aimed to evaluate hepatoprotective effect of crocin on IR injury and on microRNAs (miR-122 and miR-34a) expression.* Materials and Methods*. 32 rats were randomly divided into four groups: sham, IR, crocin pretreatment (Cr), and crocin pretreatment + IR (Cr + IR) groups. In sham and Cr groups, animals were given normal saline (N/S) and Cr (200 mg/Kg) for 7 consecutive days, respectively, and laparotomy without inducing IR was done. In IR and Cr + IR groups, N/S and Cr were given for 7 consecutive days and rats underwent a partial (70%) ischemia for 45 min/reperfusion for 60 min. Blood and tissue samples were taken for biochemical, molecular, and histopathological examinations.* Results*. The results showed decreased levels of antioxidants activity and increased levels of liver enzymes improved by crocin. The expression of miR-122, miR-34a, and p53 decreased, while Nrf2 increased by crocin. Crocin ameliorated histopathological changes.* Conclusion*. The results demonstrated that crocin protected the liver against IR injury through increasing the activity of antioxidant enzymes, improving serum levels of liver enzymes, downregulating miR-122, miR-34a, and p53, and upregulating Nrf2 expression.

## 1. Introduction

Ischemia-reperfusion (IR) injury is one of the most important etiologies of tissue damage following liver transplantation, liver resection interventions, hypovolemic shock, and trauma [1]. In spite of different therapeutic methods including pharmacology, genetic, and surgical protocols for alleviating the side effects of hepatic IR injury, it remains untreatable till now [2].

A series of physiological and biochemical changes occur following hepatic IR injury. The tissue deprivation of oxygen, nutrients, and disruption of the metabolic reactions in the ischemic phase impair mitochondrial activity which result in liver cell injury. Reperfusion in the second phase of IR exacerbates the tissue function by activating a series of events such as generation of reactive oxygen species (ROS), activation of the inflammatory, and apoptotic mediators [3].

MicroRNAs (miRNAs, miR) are a category of internal noncoding RNAs that posttranscriptionally modulate protein-coding message [4] through binding to the 3′-untranslated region (3′-UTR) of target messenger RNAs. They are 21–26 nucleotides in length that derived from precursor that cleaved by two endonucleases; Drosha and Dicer. Drosha, the nuclear enzyme for processing microRNAs, through converting primary microRNA (pri-microRNA) into a 60–70-nucleotide microRNA produces a precursor microRNA (pre-microRNA). Dicer, the cytoplasmic processor of microRNA, cleaves this precursor to generate mature microRNA [5]. Various studies showed that microRNAs play an important role in multiple physiological and pathological functions such as differentiation, development, and cancer [6].

miR-122, a 22-nucleotide microRNA, is derived from a RNA transcript from the gene* hcr* in the liver cells. It accounts for near 70% of total microRNAs pool in the liver tissue with almost 66000 copies per cell while its expression is rare in other tissues [7]. miR-122 has been shown to exert several effects in liver such as development, hepatic function [8], and hepatocyte growth [9]. It is well known that miR-122 is a noninvasive and early releasing circulating biomarker for determination of hepatic disorders [10]. A previous report showed that there is a close correlation between the serum level of miR-122 and enzymes aspartate aminotransferase (AST) and alanine aminotransferase (ALT) following warm IR-induced liver injury in rats [11]. The serum levels of liver enzymes ALT, AST, and ALP (alkaline phosphatase) have been shown to increase following hepatic IR injury [12]. These levels are the most common parameters which have been applied to manage the hepatic injury [13], while recent studies have demonstrated that miR-122 is a more sensitive parameter in hepatic injury [14, 15].

miR-34a is the another microRNA that reflects liver damage [16]. The mature miR-34, a 22-nucleotide microRNA, has three members: miR-34a, miR-34b, and miR-34c. miR-34a is a direct target of p53 [17] which inhibits cell proliferation and regulates liver function [18]. Several studies have shown that miR-34a increases during stress-induced injuries [19, 20]. It has been shown that inhibition of miR-34a protects the liver function against IR- and nonalcoholic fatty liver disease- (NAFLD-) induced injury [21]. Moreover, inhibition of miR-34a has been demonstrated to mitigate the pathological changes in other organs such as heart [19], intestine, and lung [22].

Oxidative stress, an oxidant/antioxidant imbalance [23], decreases the levels of antioxidant enzymes including catalase (CAT), superoxide dismutase (SOD), and glutathione peroxidase (GPx), via generation of ROS [24]. The antioxidant enzymes, SOD and CAT with scavenging the intracellular ROS, protect cells against IR-induced injury [25]. In addition, oxygen-derived free radicals are one of the most important causes of cellular damage following IR injury in various organs such as liver, lung, and intestine [23].

Evidences have shown that the function, stability, binding affinity, and integrity of microRNAs may be modified by ROS [26]. The redox status has also been shown to regulate the cleavage of microRNAs. Under the condition of endoplasmic reticulum (ER) stress, a transmembrane endoplasmic kinase-endoribonuclease cleaves precursor of miR-34a which in turn causes apoptosis by upregulating the apoptotic protein caspase 2 [27, 28].

p53 protein is a guardian of genome discovered for the first time in 1979 [29]. It is one of the most important tumor suppressors via inhibition of cell cycle and apoptosis [30]. One study showed that the suppression of p53 leads to a decrease in NAFLD-induced hepatic injury [31]. It is activated by multiple stimuli including DNA injury, administration of cytotoxic drugs, ROS, ultraviolet (UV) radiation, and hypoxia [29].

Nuclear factor-erythroid 2-related factor-2 (Nrf2) as a key transcription factor preserves organs versus oxidative-induced damage [32]. It is retained in the cytoplasm by keap1, released, and transferred to the nucleus for inducing transcription of antioxidant response element (ARE) which induces gene expression of its downstream targets. Nrf2 has been shown to be important for liver function. The susceptibility of liver to multiple oxidative/electrophilic stresses has been shown to increase in Nrf2 knocked out mice [33]. In addition, due to special Nrf2 target genes in liver, the metabolism and excretion of xenobiotics could be affected by this factor [34]. Evidences have shown that Nrf2 has a protective role against various liver diseases such as cholestatic liver injury [35], viral hepatitis [36], nonalcoholic steatohepatitis [37], NAFLD [38], and drug-induced liver injury [37].

*Crocus sativus* Linn. or saffron [Iridaceae family] has four major pharmacologically active constituents such as crocin, crocetin, picrocrocin, and safranal. Crocin is a water soluble carotenoid and the most important active constituent of saffron [39]. Crocin, as an antiapoptotic, anti-inflammatory, and antioxidant agent [40], has many beneficial protective effects against renal [41], gastric [42], retinal [43], and brain [44] IR-induced injuries.

The cytoprotective effects of crocin as a potent antioxidant against IR-induced tissue injuries are well established while to best our knowledge its effect on liver IR injury remained to be defined. Therefore, the present study is designed to (1) evaluate the protective effect of crocin pretreatment on IR-induced hepatic injury and (2) investigate its effect on the expression of miR-122 as well as miR-34a following hepatic IR injury.

## 2. Materials and Methods

### 2.1. Animals

Male Wistar rats (200–250 g) were purchased from the animal house of Ahvaz Jundishapur University of Medical Sciences, Ahvaz, Iran. Animals were fed on a conventional diets and tap water ad libitum. They were maintained under standard conditions of humidity, temperature (20–24°C), and 12-h light-dark cycle. Animals were deprived of food, but not water, overnight before experiments. All experiments were performed in accordance with ethics committee of Ahvaz Jundishapur University of Medical Sciences (APRC-9421).

### 2.2. Animal Grouping

In the current study 32 male Wistar rats were randomly assigned into four groups, each consisting of 8 rats. They were sham (S) group: animals received normal saline (N/S; 2 mL Kg^−1^) [41] for 7 consecutive days, intraperitoneally (ip) [45]; then laparotomy without IR induction was performed. IR group: animals received N/S with the same dose and time; then IR induction was carried out as mentioned before. Crocin (Cr) pretreatment group: Cr (200 mg Kg^−1^, ip) was given prior to intervention for 7 consecutive days [46]; then laparotomy without IR induction was done. Cr + IR group: animals received Cr with the same dose and time and was then subjected to IR induction.

### 2.3. Surgical Procedure

The rats anesthetized using a mixture of ketamine and xylazine (Alfasan Co. Woerden-Holland, 80 + 10 mg Kg^−1^, ip, respectively) [47]. Partial (70%) ischemia was induced for 45 min followed by reperfusion for 60 min as described previously [48].

At the end of experiment, rats were killed by cardiac puncture and two samples of liver tissue were taken, rinsed with N/S, snap-frozen in liquid nitrogen, and stored at −80°C. One sample was used for measurement of protein expression of p53 and another for antioxidants assay. In addition, pieces of liver were fixed in the formalin 10% solution for histopathological and immunohistochemical evaluations.

### 2.4. MicroRNAs Extraction and cDNA Synthesis

Total microRNAs were extracted from the frozen serum samples using miRNeasy/Plasma kit (QIAGEN, GmbH, Germany) according to the manufacturer's protocol. The concentration and purity of RNA were determined by spectrophotometry at wavelengths 260 and 280 nm (Nanodrop Thermo Scientific S.N:D015). The cDNA was synthesized from one microgram of the total RNA using miScript II RT Kit (QIAGEN, GmbH, Germany) according to the manufacturer's instructions.

### 2.5. Quantitative Real-Time PCR

The expression levels of microRNAs were measured by quantitative real-time polymerase chain reaction (qRT-PCR) using a Light Cycler® 96 Real-Time PCR System (Roche Diagnostics, Indianapolis, IN, USA). All PCR amplifications were performed in duplicate reactions and in final volume of 20 *µ*L containing 2 *µ*L cDNA, 10 *µ*L 2x QuantiTect SYBR Green PCR Master Mix, 2 *µ*L 10x miScript Primer Assay [miR-122 (MS00000315), or miR-34a (MS00000224); QIAGEN], 2 *µ*L 10x miScript Universal Primer [(MS0003374); (QIAGEN)], and 4 *µ*L RNAase free water using the following protocol: initial activation step at 95°C for 15 min to activate HotStar Taq DNA polymerase followed by 45 cycles at 94° for 15 s, 55°C for 30 s, and 70°C for 30 s. In addition, the no-template negative control (H_2_O) was routinely run in every PCR. The levels of microRNAs expression were normalized with RNU6 (as an internal control) and the fold change was calculated using the 2^−ΔΔCt^.

### 2.6. Protein Extraction

Frozen liver tissue was extracted using RIPA buffer (25 mM Tris-HCl pH 7.6, 150 mM NaCl, 1% NP-40, 1% sodium deoxycholate, and 0.1% SDS) containing protease inhibitor cocktail (complete mini, Roche, Indianapolis, IN, USA). To analyze the protein fraction, protein obtained using RIPA buffer, from liver samples, was resuspended in 1% SDS. The total recovery and integrity of these fractions were determined by Bradford assay and SDS-polyacrylamide gel electrophoresis.

### 2.7. Western Blotting Analysis

The extracted frozen liver proteins were separated by SDS-PAGE on 12% acrylamide gels and transferred onto a nitrocellulose membrane. The membranes were blocked with 5% nonfat dry milk dissolved in tris-buffered saline (TBS) with 0.1% Tween 20 (TBST, pH: 7.6) for 6 h and then incubated overnight at 4°C with anti-p53 antibody (mouse monoclonal, dilution 1 : 200; Santa Cruz Biotechnology; SC-100), or anti-beta actin antibody (mouse monoclonal, dilution 1 : 5000; Abcam [ab20272], USA) was added to the membrane. After 5 times of washing with TBST, membranes were incubated with a rabbit polyclonal secondary antibody to mouse IgG HRP, dilution 1 : 7000, for 90 min at room temperature. Labeled proteins were detected using a chemiluminescence western blotting system. The expression of studied proteins was semiquantified by Image J analysis software and the values were normalized to *β*-actin as a housekeeping protein.

### 2.8. Biochemical Assay of Liver Enzymes

The blood samples were centrifuged at 3000 rpm, 10 minutes, to separate the serum samples and stored at −20°C until analysis. The serum levels of liver enzymes ALT, AST, and ALP were measured using commercial kits (Pars Azmoon, Iran) according to the manufacturer's instructions by a serum autoanalyzer (BT-1500-A-A, Rome, Italy).

### 2.9. Assessment of the Activity of Antioxidants

The activity of superoxide dismutase, catalase, and glutathione peroxidase in the liver homogenates was measured using commercial kits (Zellbio GmbH, Germany) according to the manufacturer's instructions.

### 2.10. Histopathological Analysis

For histological evaluations, the liver tissues were fixed in 10% neutral formalin solution. After 72 h of fixation, tissues were dehydrated through a series of graded alcohol, embedded in paraffin, and cut into 5 *μ*m sections using a microtome (Leica RM 2125, Leica Microsystems Nussloch GmbH, Germany) and stained with hematoxylin and eosin (H&E) [49]. The images were taken and assessed using a digital research microscope (BMZ-04-DZ, Behin Pajouhesh ENG. CO., Iran).

### 2.11. Immunohistochemistry (IHC) Analysis

Formalin fixed and paraffin embedded 5 m*µ* sections of liver were applied for this procedure.

Following deparaffinization, heat-induced antigen retrieval was done through immersion of slides in pH 6.0 citrate buffer for 10–20 min [50]. Thereafter, sections were incubated with rabbit polyclonal antibody against Nrf2 (dilution 1 : 100; Abcam [ab31163], USA) and then incubated with a secondary antibody (dilution 1 : 1000; Abcam [ab6721], USA), in a moist chamber. The reaction was developed with a diaminobenzidine (DAB), sigma [D5637] chromogen solution, and slides were counterstained with hematoxylin. The images were taken and assessed using a digital research microscope (BMZ-04-DZ, Behin Pajouhesh ENG. CO., Iran).

### 2.12. Statistical Analysis

All data are shown as mean ± standard errors of the means (SEMs). For comparison among three or more groups was used one-way analysis of variance (ANOVA), followed by Dennett's or LSD post hoc tests. *P* < 0.05 was considered statistically significant.

## 3. Results

### 3.1. Crocin Pretreatment Protects the Rat's Liver against Ischemia-Reperfusion Injury

**Figure 1:**
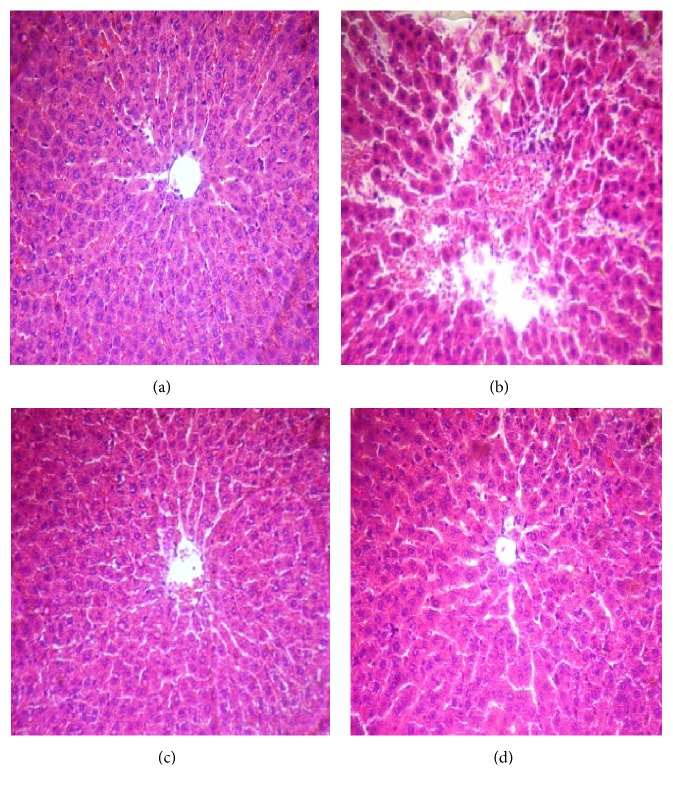
Representative microscopic images (magnification ×300) of H&E stained liver sections following hepatic IR injury. (a) Sham group shows normal appearance and there were not any histopathological changes in liver tissue; (b) IR group shows many histopathological changes including structural disarrangement of liver parenchyma, vascular congestion, central vein dilation (CV), and necrotic cells; (c) Cr group shows normal architecture; and (d) Cr + IR group, except mild dilated sinusoids, restored histopathological changes to approximately normal compared to IR group.

As shown in Figure 1, many histopathological alterations including structural disarrangement of liver parenchyma, vascular congestion, dilation of central vein (CV), and necrotic cells were evident after 45 min ischemia followed by 60 min reperfusion (Figure 1(b)). Crocin pretreatment at 200 mg/Kg for seven consecutive days effectively prevented the structural changes induced by hepatic IR injury as evidenced by mild sinusoids dilation (Figure 1(d)). As shown in Figures 1(a) and 1(c), the structure of tissue liver was normal in the sham and Cr groups.

### 3.2. Effect of Crocin Pretreatment on the Expression of Nrf2 in Liver after IR Injury

**Figure 2:**
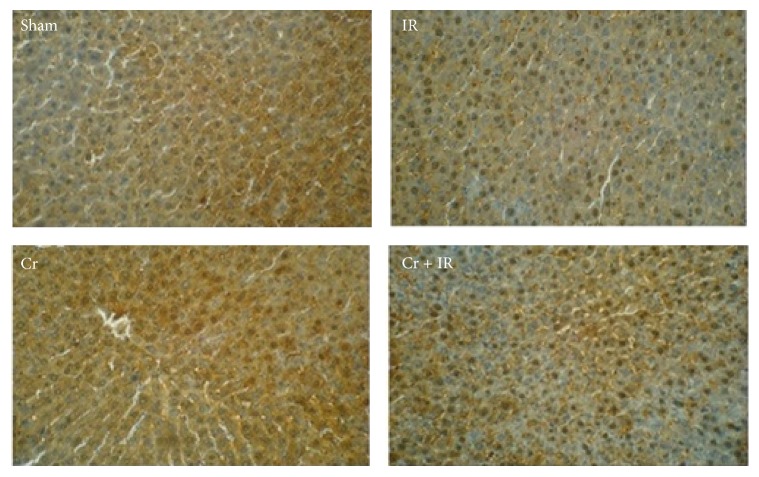
Immunohistochemical staining of liver Nrf2 expression (immunohistochemistry, ×300). Sham group: Nrf2 localized predominantly in cytoplasm of hepatocytes. IR group: Nrf2 expression decreased in cytoplasm and moderately increased in nucleus. Cr group: The immune reaction was moderate in cytoplasm. Cr + IR group: Nrf2 expression increased both in nucleus and in cytoplasm, but its expression was in nucleus higher than in cytoplasm.

As shown in Figure 2, our IHC study demonstrated that protein expression of Nrf2 in IR group decreased in cytoplasm and moderately increased in nucleus. The protein expression of Nrf2 upregulated in cytoplasm of sham and Cr groups, while in Cr + IR group the immune reaction increased both in nucleus and in cytoplasm but was in nucleus higher than in cytoplasm.

### 3.3. Effect of Hepatic IR Injury and Crocin Pretreatment on the Serum Levels of miR-122 and miR-34a

**Figure 3:**
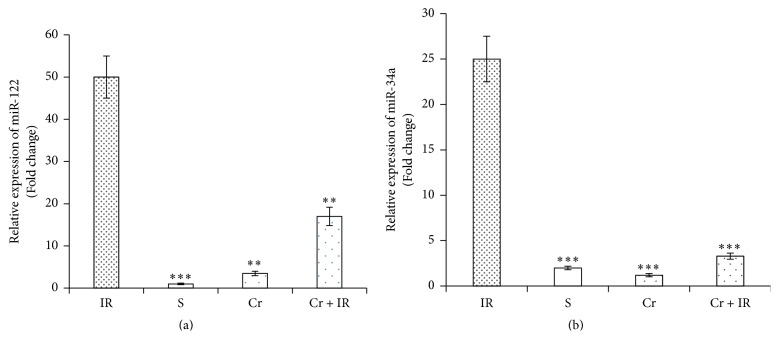
The effect of crocin pretreatment (200 mg/Kg, ip, for seven consecutive days) on the serum levels of miR-122 (a) and miR-34a (b) following hepatic IR injury. Analysis of the qRT-PCR results showed that the serum levels of miR-122 and miR-34a in Cr + IR rats were significantly lower than in IR group. Data were expressed as relative fold expression compared to IR. ^*∗∗*^*P* < 0.01 and ^*∗∗∗*^*P* < 0.001 significant difference versus the IR group. IR: ischemia/reperfusion group; S: sham group; Cr: crocin pretreatment group; and Cr + IR: animals received crocin at 200 mg/Kg (ip) for seven consecutive days prior to induction of IR.

As demonstrated in Figures 3(a) and 3(b), according to qRT-PCR results, the serum levels of miR-122 and miR-34a were significantly increased following hepatic IR injury. Crocin pretreatment (200 mg/Kg, ip, for seven consecutive days) significantly decreased these levels (*P* < 0.01 and *P* < 0.001, respectively). In the sham group, the levels of miR-122 and miR-34a were minimal (*P* < 0.001 in both cases).

### 3.4. Crocin Pretreatment Improved the Activities of GPx, CAT, and SOD Following Hepatic IR Injury

**Figure 4:**
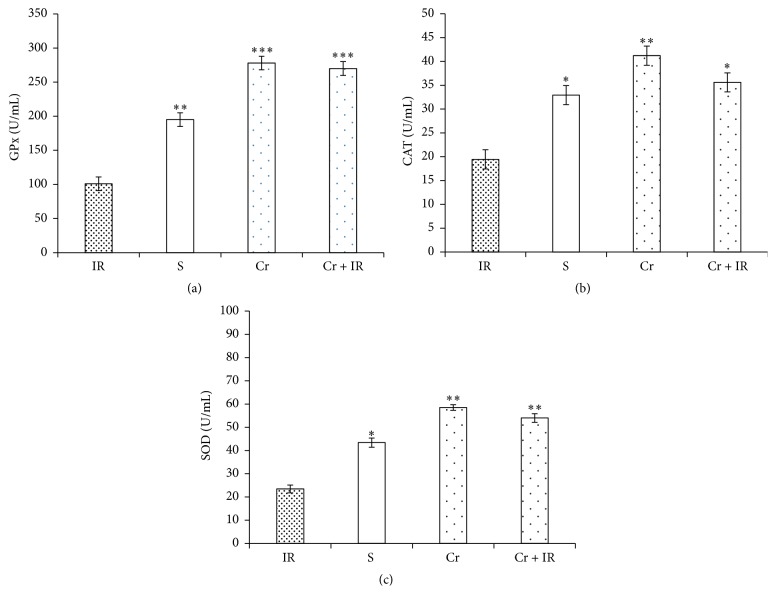
The effects of crocin pretreatment on antioxidant activity of GPx (a), CAT (b), and SOD (c) following hepatic IR injury. The levels of antioxidant activity of GPX, CAT, and SOD in IR rats were significantly lower than in sham group. Pretreatment with crocin restored these levels to normal. Results are expressed as mean ± SEM. ^*∗*^*P* < 0.05 and ^*∗∗*^*P* < 0.01 significant difference versus the IR group. GPx: glutathione peroxidase; SOD: superoxide dismutase; CAT: catalase. Cr: crocin pretreatment; IR: ischemia/reperfusion; S: sham; Cr + IR: animals received crocin at 200 mg/Kg (ip) for seven consecutive days prior to induction of IR; U/L: unit per liter. ^*∗∗∗*^*P* < 0.001.

As shown in Figures 4(a)–4(c), the activity levels of GPX, CAT, and SOD in IR group were significantly lower than in the sham group (*P* < 0.01, *P* < 0.05, and *P* < 0.05, respectively). Pretreatment with crocin (200 mg/kg, ip, seven consecutive days) restored these levels to normal. The highest levels of antioxidant activities were observed in Cr rats.

### 3.5. Crocin Pretreatment Decreased the Serum Levels of AST, ALT, and ALP Following Hepatic IR Injury

**Figure 5:**
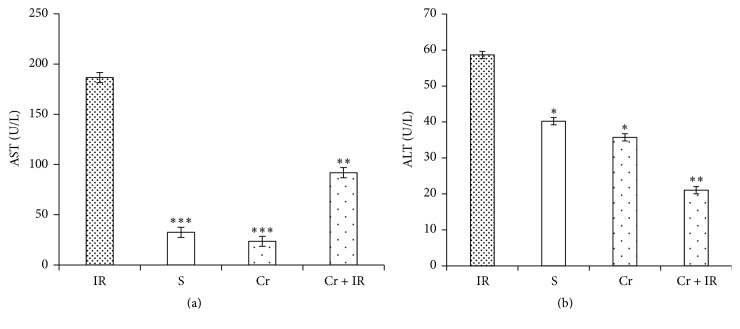
Effects of crocin pretreatment on serum levels of AST (a) and ALT (b) following hepatic IR injury. The results showed that the crocin pretreatment (200 mg/Kg, ip, seven consecutive days) decreased the serum levels of AST and ALT in comparison with IR group. Results are expressed as mean ± SEM. ^*∗*^*P* < 0.05, ^*∗∗*^*P* < 0.01, and ^*∗∗∗*^*P* < 0.001 significant difference versus the IR group. AST: aspartate aminotransferase and ALT: alanine aminotransferase. Cr: crocin pretreatment; IR: ischemia/reperfusion; S: sham; Cr +IR: animals received crocin at 200 mg/Kg (ip) for seven consecutive days prior to induction of IR; U/L: unit per liter.

**Figure 6:**
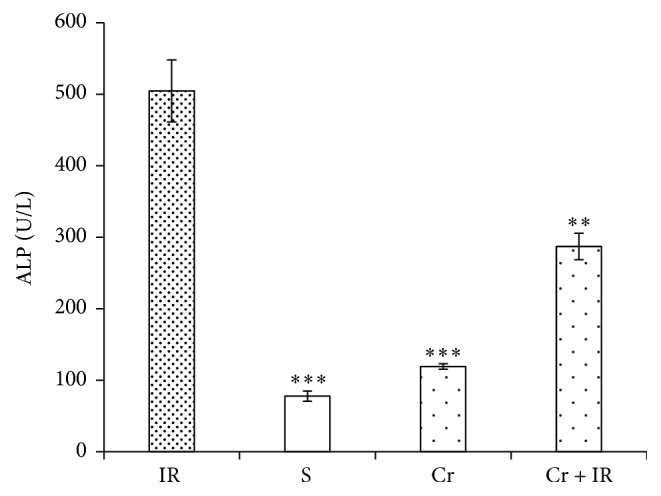
Effects of crocin pretreatment on serum level of ALP following hepatic IR injury. The results showed that the crocin pretreatment (200 mg/Kg, ip, seven consecutive days) decreased the serum levels of ALP in comparison with IR group. Results expressed as mean ± SEM. ^*∗∗*^*P* < 0.01 and ^*∗∗∗*^*P* < 0.001 versus IR group. ALP: alkaline phosphatase; Cr: crocin pretreatment; IR: ischemia/reperfusion; S: sham; Cr + IR: animals received crocin at dose 200 mg/Kg (ip) for seven consecutive days prior to induction of IR; U/L: unit per liter.

Figures 5(a) and 5(b) show that following IR injury, the serum levels of AST and ALT in IR group were significantly higher than in the sham group (*P* < 0.001 and *P* < 0.05, respectively). These levels in Cr + IR group were significantly lower than in the IR group (*P* < 0.01 in both cases). As illustrated in Figures 5(a) and 5(b), there were no differences between the serum levels of AST and ALT in Cr and sham groups. The results also demonstrated that 45 min ischemia followed by 60 min reperfusion increased the serum level of ALP (Figure 5(c)). This level in sham group was significantly lower than in IR group (*P* < 0.001). Pretreatment with crocin (200 mg/Kg, ip, seven consecutive days) significantly reduced this level to near normal (*P* < 0.01) (Figure 6).

### 3.6. Crocin Pretreatment Downregulated the Protein Expression of p53 Following Hepatic IR Injury

**Figure 7:**
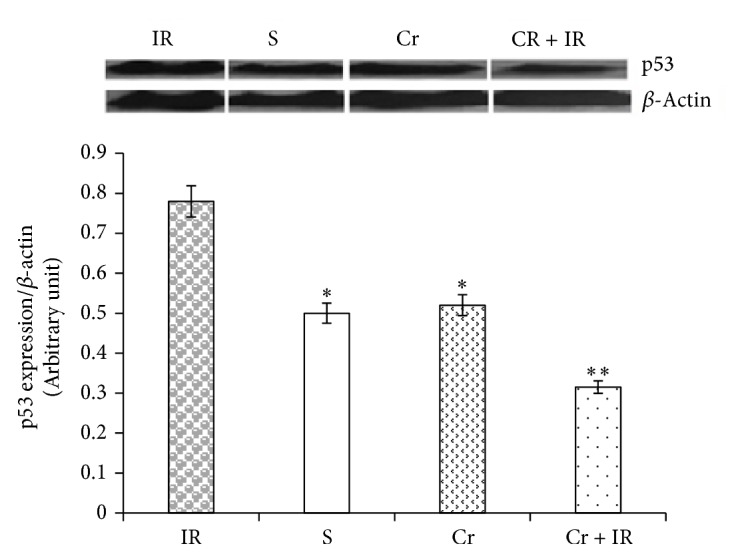
Effects of crocin pretreatment on protein expression of p53 following hepatic IR injury. Analysis of western blot results showed that the level of protein expression of p53 in IR group was significantly higher than in sham, Cr, and Cr + IR groups. Crocin pretreatment significantly decreased this level to near normal. Results expressed as mean ± SEM. ^*∗*^*P* < 0.05 and ^*∗∗*^*P* < 0.01 versus the IR group. Cr: crocin pretreatment; IR: ischemia/reperfusion; S: sham; Cr + IR: animals received crocin at 200 mg/Kg (ip) for seven consecutive days prior to induction of IR.

As illustrated in Figure 7, the level of protein expression of p53 in IR group was significantly higher than in sham, Cr, and Cr + IR groups. Pretreatment with crocin (200 mg/Kg, ip, seven consecutive days) prior to induction of IR significantly reduced this level to near normal in comparison with IR group.

## 4. Discussion

The present research showed that crocin (Cr) protects the rat's liver against IR injury. The results of the current study showed that crocin (a) decreased the increased serum levels of miR-122 and miR-34a in rats following IR-induced injury; (b) downregulated the protein expression of p53 in the liver; (c) decreased the increased serum levels of liver enzymes; (d) increased the decreased level of antioxidant activity of SOD, GPx, and CAT in the liver; (e) mitigated the histopathological changes induced by hepatic IR injury; and (f) increased Nrf2 expression.

The serum level of hepato-specific miR [miR-122] has been shown to increase faster than the serum concentrations of ALT and AST enzymes following hepatic IR injury [51]. Our qRT-PCR results showed that the serum level of miR-122 enhanced after IR-induced liver injury. The present findings also showed that crocin pretreatment decreased the overexpression of miR-122 induced by IR injury. There is not any report about the effect of crocin on serum levels of miR-122. Therefore, it seems that this effect of crocin pretreatment on the level of miR-122 expression could be secondary to its cytoprotective activity as demonstrated by histopathological findings.

The present histopathological findings showed that pretreatment with Cr effectively but not completely prevents deleterious effect of IR-induced injury in the rat liver. The qRT-PCR results showed that the serum levels of miR-122 in Cr + IR rats were significantly decreased but it was still higher than that in the sham group. Our results also showed that the highest and lowest levels of miR-122 were, respectively, observed in IR and sham groups. As evidenced by the current histopathological and qRT-PCR results, there is a close correlation between the serum level of miR-122 and severity of liver tissue damage. Therefore, these findings taken together suggest that the monitoring of the serum level of miR-122 could be a high sensitive, early, accurate, and reliable biomarker for determining the progression of liver injuries.

The present qRT-PCR results also showed that the serum level of miR-34a increased following hepatic IR injury. Bader has demonstrated that miR-34a elevated after IR-induced liver injury in rats [52]. It has been shown that the expression of miR-34a is involved in age-related loss of antioxidant defense system in the liver [53]. Moreover, the involvement of miR-34a in age-related loss of antioxidant defense system has been reported [54]. Nrf2 plays an antioxidant agent by regulating the gene expression of antioxidant enzymes [21]. A study showed that the activity of Nrf2 and its ARE target in liver is inhibited by miR-34a [54].

Our results showed that there is an inverse correlation between the serum level of miR-34a and Nrf2. The highest level of miR-34a and lowest level of Nrf2 were observed in IR group. In addition, the present results also showed that there is a direct correlation between antioxidants activity and the level of Nrf2 expression. These results together suggest that miR-34a by inhibiting the activity of Nrf2 and its downstream target genes decreased the activity of antioxidants. ROS have been documented to affect the expression of microRNAs in an in vitro experiment [55, 56]. Therefore, the increased production of ROS following IR injury [57] could be the other mechanism caused to the overexpression of miR-34a.

Therefore, on one hand, the accumulation of intracellular ROS by hepatic IR injury as evidenced by a previous study [57] and, on the other hand, the reduction of antioxidant activity as shown by the present results both contribute to the tissue damage in the rat's liver.

The current results also showed that crocin pretreatment decreased the expression levels of miR-34a and increased the activity of antioxidant enzymes.

As mentioned earlier, the level of Nrf2 expression decreased following hepatic IR injury. This level increased by crocin pretreatment. Our findings are in agreement with an in vitro study which showed that crocin increases the expression and activity of Nrf2 in Hela cells [58]. Recently, in one study, authors have shown that crocin pretreatment protects the gastric mucosa against IR-induced injury through upregulating the mRNA expression and activity of antioxidant enzymes in rats [59]. The present results also showed that the activity of all studied antioxidant enzymes [SOD, CAT, and GPx] increased after crocin pretreatment. These findings together suggest that the promotion of Nrf2 by crocin decreased IR-induced ROS accumulation which led to downregulating the expression of miR-34a.

It has been shown there is a positive correlation between p53 and miR-34a via SIRT1-p53 pathway which regulates apoptosis [60]. Another mechanism contributing in apoptosis is Let-7/CD95/p53/miR-34a pathway [61]. Additionally, it was identified that induction of miR-34a as a fine tuning of gene expression by p53 significantly leads to changes such as apoptosis and regulation of cell cycle [62]. On the other hand, a study indicated that chemical suppression of p53 inhibits miR-34a upregulation and dramatically decreased oxidative stress, apoptosis, and hepatic steatosis [31].

Our study also demonstrated that crocin pretreatment decreased the overexpression of p53 and miR-34a following hepatic IR injury. Therefore, these findings suggest that crocin similar to carnosic acid [63] and hydrogen sulfide [54] through silencing the expression of miR-34a protects the rat liver by inhibiting apoptosis against IR-induced cell injury. P53 [a stress-related transcription factor] by activating Drosha facilitates the processing of pri-miR-34a to pre-miR-34a. Therefore, p53 increases the expression level of miR-34a [64, 65]. These reports suggest that the lower expression of miR-34a in crocin pretreated rats could be due to the inhibitory effect of crocin on p53 expression.

It has been shown that the serum levels of AST and ALT increase following IR-induced liver injury [66, 67]. In addition, ALP as a performance parameter of liver injury has been shown to elevate after drug-induced hepatotoxicity [68]. These enzymes were released from hepatocytes and elevated in serum during necrosis, structural damage, or cellular leakage [13]. Crocin decreases the concentrations of these enzymes following drug-induced liver damage [13]. The present findings showed that crocin pretreatment decreased the increased serum levels of these enzymes. As seen in Figures 4(a)–4(c), the highest enzymatic antioxidants activity was seen in crocin pretreatment rats. These results showed that administration of crocin as a potent antioxidant for a week effectively enhanced the antioxidant capacity in liver tissue.

Tumor protein 53 [p53] is a guardian of genome induced by DNA damage, ROS, hypoxia and overexpression of oncogenic agents that lead to cell cycle arrest and apoptosis [24, 69]. One survey showed contribution of free radicals in p53 activation and eventually apoptosis [70]. Our results showed that the expression of p53 increased following IR injury. The findings of the present study suggest that this increase could be in part due to attenuation of the antioxidant activities secondary to ROS accumulation.

Crocin has been reported to protect the rats liver against cisplatin-induced toxicity through downregulating the protein expression of p53 [24]. In agreement, our western blot result showed that the IR-induced overexpression of p53, effectively prevented by crocin pretreatment. Therefore, crocin through downregulating the expression of an apoptotic protein [p53] protected the rats liver against IR injury. What is the clinical implication of the present results? The findings of the present study showed that crocin as a potent antioxidant through downregulating miR-122 and miR-34a effectively controls ROS-induced liver injury. Miravirsen has been shown to treat HCV infection through functionally inhibiting miR-122 [71]. Therefore, targeting microRNAs by reducing their expression as performed by crocin in this research and also carnosic acid and hydrogen sulfide in previous reports or inhibition of them as showed by Suzuki et al. [71] both can be effective for treating the liver disorders.

## 5. Conclusion

The results of the present study showed that crocin pretreatment (1) protected the rat's liver against hepatic IR-induced injury; (2) upregulated the protein expression of Nrf2; (3) downregulated the expression levels of miR-122 and miR-34a; (4) improved the liver enzymes AST, ALT, and ALP; (5) increased the antioxidant activity of SOD, GPx, and CAT; and (6) decreased the protein expression of p53 following hepatic IR-induced injury.
